# Simulation of sediment transport in Krueng Baro River, Indonesia

**DOI:** 10.4102/jamba.v12i1.934

**Published:** 2020-11-16

**Authors:** Hairul Basri, Azmeri Azmeri, Wesli Wesli, Faris Z. Jemi

**Affiliations:** 1 Department of Soil Science, Faculty of Agriculture, Universitas Syiah Kuala, Banda Aceh, Indonesia; 2 Department of Civil Engineering, Faculty of Engineering, Universitas Syiah Kuala, Banda Aceh, Indonesia; 3 Department of Civil Engineering, Faculty of Engineering, Universitas Malikussaleh, Lhokseumawe, Indonesia; 4 Department of Electrical Engineering, Faculty of Engineering, Universitas Syiah Kuala, Banda Aceh, Indonesia

**Keywords:** sediment transport, Krueng Baro River, Keumala weir, sedimentation, soil science

## Abstract

An alluvial river system is a dynamic system that can alter the hydraulic flow and sediment load. Sediment modelling can enhance our understanding of river morphology. This model has also proved to be a useful tool to overcome problems concerning sediment transport and sedimentation and to provide effective mitigation measures. This article discussed the concept of sediment modelling. The sediment transport simulation method used sediment river hydraulic – two dimension (SRH-2D), which is a hydraulic model for river systems developed by United States Bureau of Reclamation (USBR). The SRH-2D results showed that the Krueng Baro River underwent many sedimentations, and the increase in the height of the layers was observed during the simulation period. It examined the main categories of sediment transport models and presented the boundaries. Besides, this study also considered the constraints on the operation of Perusahaan Daerah Air Minum (Indonesian regional water utility company) (PDAM) and irrigation intakes because of the sedimentation in the Krueng Baro River flow, taking into account the structure of the Keumala weir. Specifically, this study thoroughly discussed the position and altitude of river aggradation and degradation to minimise conflict of interest between the PDAM and irrigation water supply because of sedimentation.

## Introduction

Natural and human factors have an impact on rivers. Rivers undergo dramatic changes in the long term, leading to geomorphological changes. The influencing factors include severe erosion on bed or banks, sectional movements and sedimentation (Haghiabi & Zaredehdasht 2012). An investigation of river morphology describing the river geometry, riverbed shape, longitudinal profiles, cross-sections and river shape change is one of the main topics in river engineering (Graf 1984; Van Rijn 1993).

All rivers generally contain sediment loads, depending on the nature of the watershed, including the topography, land cover, land use and soil type. Flow energy is also an essential factor influencing the concentration of sediment loads in rivers. Sedimentation in rivers is a significant problem as rivers comprise large amounts of sediment (Omran & Jaber 2017). The sediment loads in the river increase in rainy season because of the increased flow rate. Rainfall also raises sediment transport through watershed run-off, especially at the onset of the rainy season (Mohammad et al. 2016). Problems related to river use are unavoidable, especially aggradation and degradation. Aggradation reduces the water reservoir in the river leading to floods, whilst degradation causes damage to river structures because of the riverbed decline. Therefore, analysis of river sediment transport is paramount to optimise the river handling (Rafsanjani 2017).

Sediment will reduce the water velocity in the river so that the suspended sediment is deposited. When sediments accumulate in the river, the river gradually loses its ability to transport water. Over time, this deposition will reduce the river volume (Kheder et al. 2014). River sedimentation is the most frequent technical challenge faced by irrigation systems (Ochiere, Onyando & Kamau 2015). Thus, the importance of effective sediment management in rivers from an economic, social and environmental perspective is increasingly growing (Unesco/Irtces 2011).

The process of sediment transport and sedimentation can change the riverbed topography (Holmes 2010), and the sedimentation is a key factor hindering river development and management. Besides, the increasing human activity, such as extensive watershed damage, has increased deposited sediments in rivers.

When the flow enters the weir or reservoir, the velocity and energy of the flow decline, causing the sediment to settle. A weir is a building serving to increase the water-level elevation in the river to fulfill the water needs such as irrigation, clean water, electricity generator and flood control (Azmeri et al. 2017). Coarse sediments are usually deposited at the beginning of the weir, whilst fine particles are settled in the direction of water flow in the weir or reservoir (Mohammad et al. 2016).

In the last few decades, the relationship between geomorphology and river engineering remains unclear and inappropriate. River engineering may be required to use regular geometric and prismatic cross-sections; various modifications and stabilisation structures throughout the river and flow control. Many previous studies showed the importance of morphology in river engineering.

Krueng Baro River is one of the vital rivers in Indonesia. This river is a water source irrigating 11 950 ha of irrigated land in the Krueng Baro scheme and supplying clean water needs for the people in the downstream. However, the Krueng Baro River has a considerable load because of its long path passing through various geological formations that produce high river concentrations. The high concentration causes more erosion in the riverbed and river walls as well as increases river sediment load. In addition, there is an intake of PDAM Tirta Mon Krueng Baro located around ± 78 metres from the upstream of Keumala weir. The high sedimentation at the PDAM intake contributes to the frequent disruption of the water supply to the PDAM and creates a conflict of interest between clean water and irrigation water supply. Thus, sediment management is required to increase water supply for the irrigation scheme and clean water (Kisi 2012; Kuscu, Bölüktepe & Demir 2009). Hydraulic and operational performance analysis is crucial in the river system without lining, where sedimentation is frequently encountered (Rijo & Arranja 2005). With better details of water intake, reservation and distribution sedimentation analysis could help to identify the constraints in hydraulic performance to find the alternatives for improving the circumstances (Oh et al. 2016). This study aimed to analyse the hydrodynamics and sediment transport of the Krueng Baro River that occurred in the upstream and downstream of Keumala weir. This research contributes to provide valuable information on the shape of the river cross-sections. It also presents data concerning the average water depth in the upstream and downstream of the Keumala weir at the Krueng Baro River. Therefore, the sediment rate in the Krueng Baro River can be handled appropriately.

## Data and methods

**FIGURE 1:**
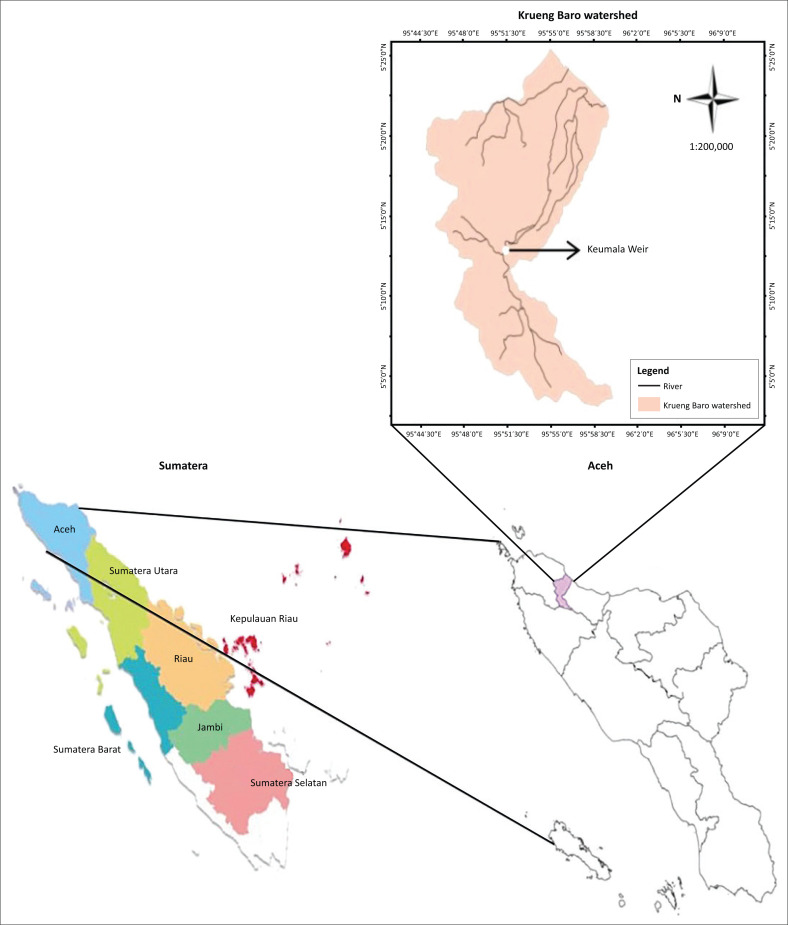
The study area – Keumala Weir at Krueng Baro River.

This research was conducted at Keumala weir of the Krueng Baro River in Pidie District, at the coordinates of 5°13’10.2 “N and 95°51’38.8” E (Figure 1). The research site was precisely from the upstream to the downstream of Keumala weir, approximately 1 km.

### Data and method used

This research required primary data including location survey, sediment sampling, hydrometry in the upstream and downstream of Keumala weir as well as the hydrodynamic analysis and sediment transport rates analysis. The stages undertaken in this study were as follows:

- Bedload sediment sampling was undertaken at three points using the Grab Sampler, namely the left, the right, and the centre of the river. The procedure of floating sediments sampling adhered to the Guidelines of Construction, Civil Building Survey and Reservoir Sedimentation Monitoring (2009).
- Examining the bedload sediment samples. The test involved grain size analysis in determining the soil properties (size and soil types). In addition, specific gravity test was conducted to determine the mass density of soil grains or soil specific gravity. Both tests were undertaken at the Soil Mechanics Laboratory in the Department of Civil Engineering, Faculty of Engineering, Syiah Kuala University.
- Hydrometry and Calibration model.

Hydrometry is measuring the river flow velocity using a current meter with the propeller diameter of 80 mm and the pitch of 125 mm. The current meter used in this study is a digital device directly displaying the results of flow velocity in each section. The flow velocity measurement was conducted on a 4-meters transverse section. The sediment sampling was also undertaken in this location. The measurement results were then used as the calibration of hydrodynamic modelling. The measurement results of the river topography originated from Balai Wilayah Sungai Sumatera I (2017). These are in the form of a longitudinal section and a cross section. There were 20 measurement points of the transverse cross-sections from the upstream to the downstream, as marked by KB-0 to KB-19. Bathymetry data were generated from the measurements of 500 metres towards the upstream and the downstream of the Keumala weir. Rectangular shaped mesh (Patch-SMS) was used to obtain accurate results.

- Boundary conditions were required in both upstream and downstream models.
  - The upstream boundary condition, inflow discharge and sediment supply rate are as follows:
    - Series I: The upstream boundary condition was an average discharge for 1 year based on data from Sumatra River Region I, 12.99 m^3^/second.
    - Series II: The upstream boundary condition was the unsteady inflow of flood discharge for the Q25 return period plan, 794.38 m^3^/second.
    - The level of sediment supply is assumed to be equal as the transport capacity for non-cohesive sediments.
  - The downstream boundary condition
    - Series I: Downstream boundary condition was the water-level elevation +87.86 metres
    - Series II: Downstream boundary condition was in the rating curves.
- Another modelling parameter includes the specific gravity for non-cohesive sediments of the upstream and downstream weir (2706 tons/m^3^ and 2659 tons/m^3^, respectively). The specific gravity values of these sediments were generated from the laboratory test results of the Krueng Baro riverbed sediments of the upstream and downstream of the weir.

### Sediment transport analysis

Sediment river hydraulic – two dimension (SRH-2D) is a hydraulic model for river systems developed by USBR by merging the structural features of the flow and is better than the previous version. The SRH-2D regulator equation is a 2D model of the average depth, hydraulic transport and sediment model for river systems. Sediment transport and riverbed dynamics were analysed using the approach of Greimann, Lai and Huang (2008), classifying non-uniform sediments into several sediment size-classes (N_Sed_). Each size-class of k in the water column was controlled by the non-equilibrium mass conservation equation, as follows:

$$\begin{array}{l} \frac{\partial hC_{k}}{\partial_{t}}+\frac{\partial \cos(\alpha_{k})\beta_{k}{\text{v}}_{\text{t}}\text{h}C_{k}}{\partial x}+\frac{\partial \sin(\alpha_{k})\beta_{k}v_{t}hC_{k}}{\partial y}\\ =\frac{\partial }{\partial x}\left(f_{k}D_{x}\frac{\partial hC_{k}}{\partial x}\right)+\frac{\partial }{\partial y}\left(f_{k}D_{y}\frac{\partial hC_{k}}{\partial y}\right)v_{k} \end{array}$$ [Eqn 1]

where k = the sediment class-size; *C*_*k*_ = the average depth of sediment concentration based on volume; velocity ratio *β_k_= V_sed,k_*/*V_t_* represents sediment velocity ratio to the flow; $V_{t}=\sqrt{U^{2}+V^{2}}$ = the average total flow velocity depth, *α_k_* = the angle of sediment transport direction to the x axis; *f_k_* = the parameter represents the percentage of sediment in suspension (1.0 for floating sediment and 0 for coarse sediment); *D*_*x*_ and *D*_*y*_ = the coefficient of mixing sediments in the *x* and *y* axis direction.

The SRH-2D equation was solved by employing the numerical method using the average depth (Lai 2010). All regulator equations generally use the volume method to the local and global mass conservation with implicit time scheme. The sediment transport Equation (1) was discrete in the same way as to flow equation. The sediment depth *hC*_*k*_ was the main dependent variable and fractional step method adopted (Yanenko 1971).

## Results and discussion

### Hydraulic calibration of model

Based on the hydrometric measurements, the flow velocity was identified and then used to calculate the discharge flow, 20.74 m^3^/s. The Manning’s coefficient is a calibration parameter in this modelling. In line with the research conducted by Hameed and Ali (2012), the calibration was done by selecting the appropriate Manning (n) coarseness coefficient. The conformity between the model and the observed data was within a reliable accuracy. The closeness between the flow depth in the modelling and the hydrometry showed a Manning’s coefficient of 0.04.

### Bedload sediment

The coarse particles moving along the riverbed are generally known as bedload sediments. The movement of the riverbed particles indicates its presence. The movement includes shifting, rolling or jumping, but they are never detached from the riverbed. The movement can sometimes reach a certain distance, as evidenced by the mixing particles moving to the downstream (Holmes 2010).

#### Series I modelling results

Series I Modelling had the upstream boundary condition of the average discharge with 5 days simulation duration. It aimed to illustrate the long-term sedimentation and erosion rate under a normal discharge condition. Discharge information was required to calculate the sedimentation rates. Mokonio (2013) argued that one of the factors influencing the sedimentation process is discharge flow. When the discharge flow is low, the sediment transport is small. Inversely, when the discharge flow is high, the river can carry higher sediment loads with a broader range of sediment sizes. However, in reality, the discharge carries varying sediment load. In some rivers, the ratio of the maximum and minimum discharge can be up to 1000 or more (Garde & Ranga Raju 1977). The diverse variety of the river flow becomes the challenge in selecting a representative discharge to study the river flow characteristics. Some researchers proposed different methods for choosing a representative discharge (Garde & Ranga Raju 1977), one of which is the average discharge.

The flow pattern and the velocity vector information shows that the water velocity in the left upstream of the weir (I) was significantly higher than the right upstream of the weir (II). Some flow went to the downstream of the weir with the similar velocity because of the bend (III), whilst others changed its direction to a rotating flow with the low velocity, less than 0.5 m/s. On the other hand, in the downstream of the weir, the large magnitude velocity vector was precisely below the crest of the weir, a supercritical flow. There was an increase in the flow at the location (IV), as a result of a drastic change of the bed channel slope. With the average discharge flow of 12.99 m^3^/s, the flow only flowed to the left of the weir, resulted in no flow in the intake location of PDAM (II).

**FIGURE 2:**
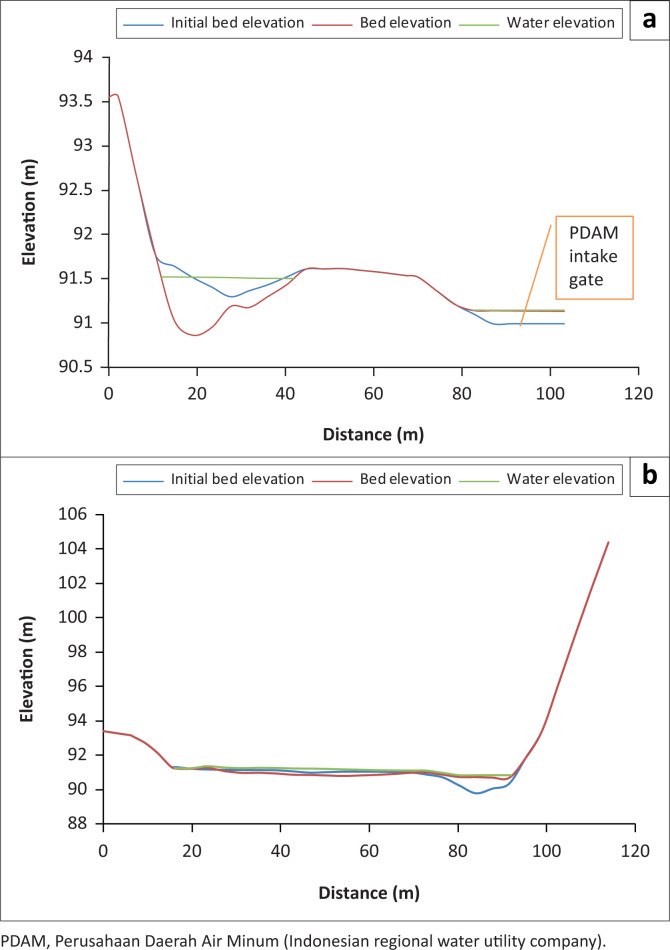
Water profile of sections C and D on day 4. (a) water profile of section C on day 4; (b) water profile of section D on day 4.

As Series I modelling was steady-state modelling, the water-level elevation and depth were relatively constant. The change in water-level elevation during the model simulation was merely the change from the initial condition to a stable flow. The water profile, the existing bed elevation and the bed elevation are shown in C and D transversal sections, which were the PDAM and the irrigation intake, respectively (Figure 2).

The riverbed elevation at the PDAM intake increased by 16 cm, which is higher than the initial bed elevation. In line with the velocity vector, the PDAM (II) intake location had no flow. The similar condition almost occurred at the irrigation intake location. However, as the type of the irrigation intake was a bottom outlet, the irrigation water could still flow into the carrier channel.

**FIGURE 3:**
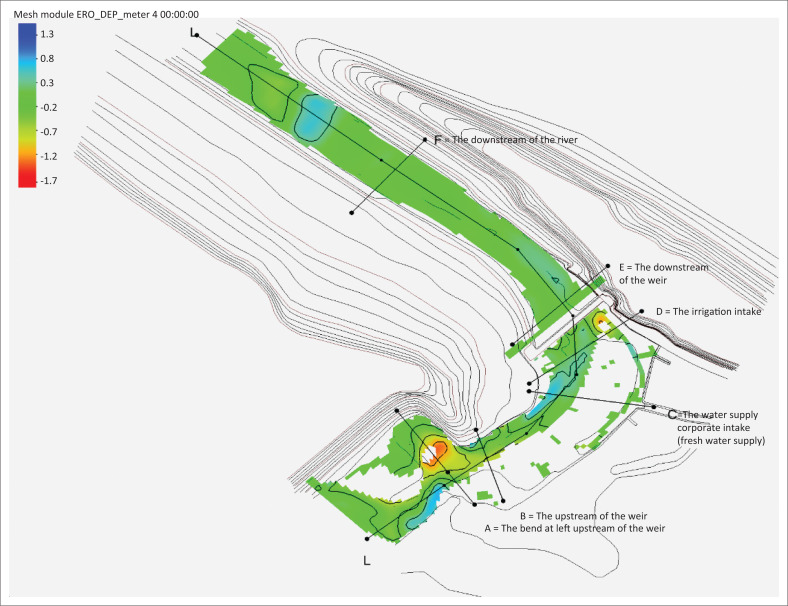
The sediment transport pattern of series I modelling.

**FIGURE 4:**
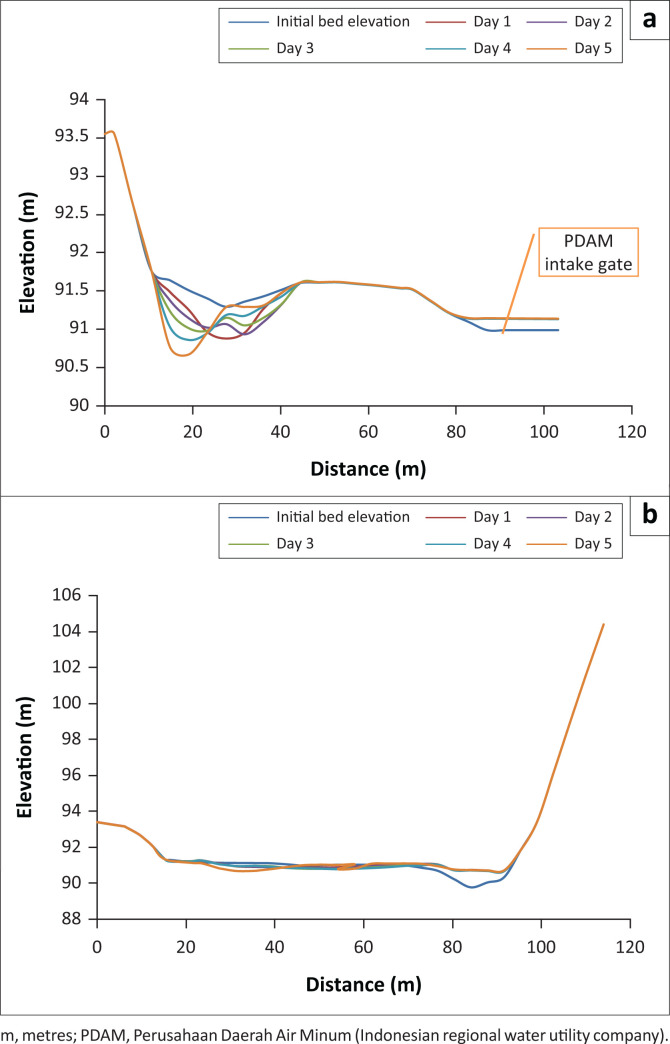
The bed elevation profile of sections C and D on days 1, 2, 3, 4 and 5. (a) bed elevation profiles of section C on days 1, 2, 3, 4 and 5; (b) bed elevation profiles of D section on days 1, 2, 3, 4 and 5.

Figure 3 presents the sediment transport pattern of Series I modelling, whilst Figure 4 displays the scour depth contour (+ for erosion and – for sedimentation) on days 1, 2, 3, 4 and 5, specifically in the PDAM and irrigation intakes.

Generally, there was a scour (I) at the left upstream of the weir because of the concentrated flow on the left; it is in line with the flow velocity. As a result of the bend in Location III, the beginning of the bend before the weir (approximately 200 metres from the upstream of the weir), a rotating flow with low velocity occurred causing aggradation (sedimentation). In addition, at Location I, the location of the inner bend, right before the weir (approximately 50 metres from the upstream of the weir), rather deep degradation (erosion) occurred. With the average annual discharge of 12.99 m^3^/s, sedimentation occurred in Location II, where the PDAM intake is located, but it was not too large. This location was covered by sediments as high as 16 cm at 10:00 on day 1. Sediment concentration occurred in the irrigation intake. Overall, during the 5 days simulation period, the total volume of water entering the volume control was 5 611 500 m^3^ whilst a total input volume of sediments was 312 100 m^3^.

#### Series II modelling results

Series II modelling had the upstream boundary condition of unsteady discharges in the form of a Q25 flood discharge plan. This model aimed to obtain the pattern of erosion and sedimentation during storm water.

Almost similar to Series I modelling, the flow pattern and velocity vector shows that because of the bend (III) some flow went to the downstream of the weir by similar velocity. On the other hand, some flows changed their direction, becoming a rotating flow with a flow velocity lower than the main channel. The water velocity at the left upstream of the weir (I) was much larger than the right one (II). In the downstream of the weir, there was a large magnitude of velocity vector right under the crest of the weir, a supercritical flow. There was an increase in flow at Location IV because of the drastic change of the bed channel.

**FIGURE 5:**
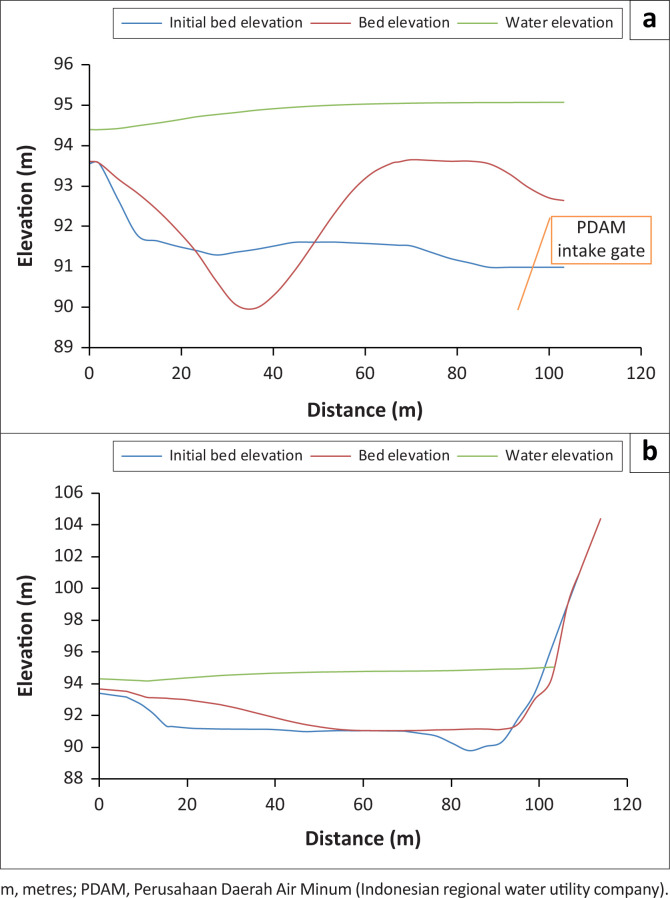
Water profile of sections C and D at 10:00 on day 1. (a) water profile of section C at 10:00 on day 1; (b) water profile of section D at 10:00 on day 1.

Series II modelling was in an unsteady condition, so the water elevation and depth changed as the time change of water elevation and depth. The peak of flood hydrograph was at the 7th hour, with a peak discharge of 794.38 m^3^/s. At the 5th hour, the flood discharge had reached up to 563.85 m^3^/s, and thus the water profile was already high at the 5th hour. A similar case happened at the 10th hour. At the 20th hour, the flood discharge was only 267.37 m^3^/s, so that the water accumulated in the lower part. The water profile, existing bed elevation and bed elevation are presented in C and D transversal sections (Figure 5). They were the PDAM and irrigation intake accordingly.

**FIGURE 6:**
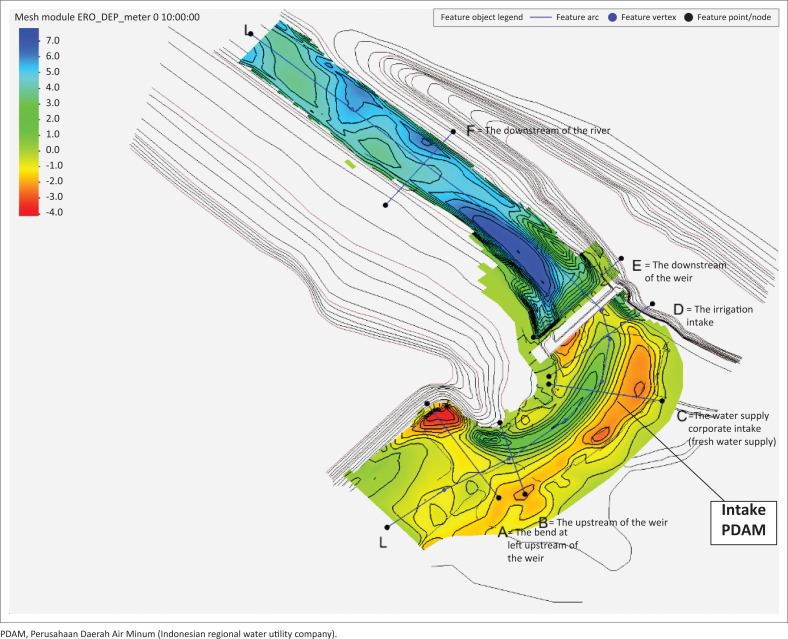
Sediment transport pattern of series II modelling.

**FIGURE 7:**
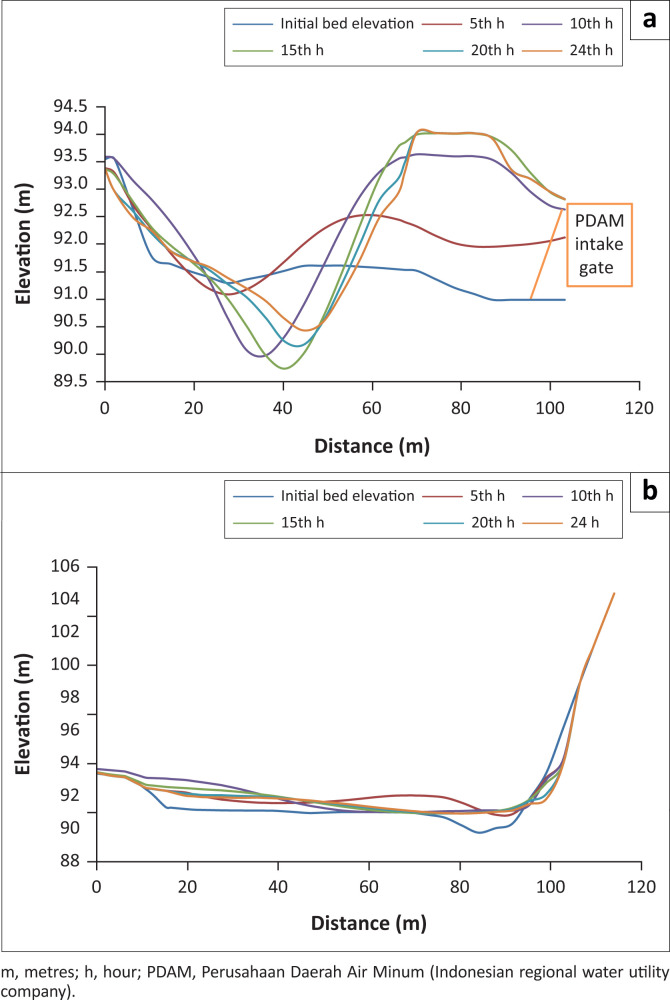
Bed elevation profile of sections C and D at 5, 10, 15, 20 and 24 h. (a) bed elevation profile of section C at 5, 10, 15, 20 and 24 h; (b) bed elevation profile of section D at 5, 10, 15, 20 and 24 h.

Figure 6 displays the sediment transport pattern of the Series II modelling and Figure 7 presents the scour depth contours (+ for erosion and – for sedimentation) at the 5th, 10th, 15th, and 20th h, especially in PDAM and irrigation intakes.

As a result of the concentrated flow on the left upstream, scour (I) occurred on the left upstream of the weir, the location of the inner bend, just before the weir (about 50 metres from the upstream of the weir). The scour was consistent with the flow velocity. A low velocity rotating flow which led to aggradation (sedimentation) occurred at Location III because of a bend. The initial location of the bend was before the weir (approximately 200 metres from the upstream of the weir). Sedimentation also took place at the right side of the weir (II), at the location of PDAM intake. The intake in this location was covered by 1 m sediment at the 10-h simulation on Day 1. This sedimentation at the PDAM intake required immediate treatment to avoid the reduction of water supply to the Keumala PDAM. In addition, sedimentation also occurred at the irrigation intake.

There was a vast degradation at the downstream of the weir (IV), influenced by the amount of water discharge through the weir. This problem required to be resolved immediately by fixing the damaged stilling basin and building a massive stilling basin. Overall, the total volume of water entering the volume control was 34 858 000 m^3^ for 2 days (48 h) simulation, with a total input of sediment volume of 624 170 m^3^.

The results of sediment transport during the flood peak are presented in Figure 7. The results of the 2D-SRH model clearly showed that the shallow areas, such as near the floodplain area, had more sediments than the main river channel. This finding is in line with the research of Mwanuzi and De Smedt (1997). The downstream weir showed less deposition, meaning sediments required a longer time to travel to the downstream.

Each change in the flow and dimension because of the structure will modify the regime of the river; it will trigger the degradation and aggradation leading to the new regime. Degradation and aggradation will continue to occur until the sediment transport capacity is reached. When the discharge decreases, the sediment starts to release and settle in the downstream. The existence of weir influences the patterns of water and sediment discharge. The water discharge released from the weir may be smaller or equal to the initial river discharge, but the sediment discharge released from the weir may be close to zero. The water retained in the weir changes the sediment transport capacity by limiting the free movement of sediments in the weir. However, when the flow is strong enough, particles are delivered to the downstream of the river.

Sedimentation in the area surrounding the Keumala weir body is a serious matter. Some of the sedimentations can compromise the function of the weir and the infrastructure around it, causing the closure of the Keumala PDAM intake and disrupts the irrigation intake. The reduced water supply to each intake causes a conflict of interest between both intakes. To overcome this problem, sediment dredging and transporting should be conducted. After the dredging and transporting of sediments has been undertaken, the weir can run optimally to fufill the needs of irrigation and PDAM water.

## Conclusion

The results of the study indicate that the SRH-2D model has been successfully applied to the Krueng Baro River. The SRH-2D results showed that the Krueng Baro River underwent many sedimentations, and the increase in the height of the layers was observed during the simulation period. The findings of this research highly recommend riverbed dredging in the areas with erosion-prone sedimentation and stabilisation. It is important to note that even though the sediment transport function of this study is valid it may not provide higher accuracy results in other cases. This is because each sediment transport function is subject to the specific geometry, hydraulics and sedimentation, and thus it is unable to be generalised to various other circumstances. Therefore, the geometry, hydraulic and sedimentation conditions of each sediment transport function and the river scope of the research are critical in the application of this method.
